# Clinical characteristics and prognostic analysis of anti-gamma-aminobutyric acid-B (GABA-B) receptor encephalitis in Northeast China

**DOI:** 10.1186/s12883-019-1585-y

**Published:** 2020-01-03

**Authors:** Xinyue Zhang, Yue Lang, Lichao Sun, Weiguanliu Zhang, Weihong Lin, Li Cui

**Affiliations:** 1 Department of Neurology, Neuroscience Center, The First Hospital of Jilin University, 71 Xinmin Street, Changchun, 130021 Jilin China; 2 Department of Emergency, The First Hospital of Jilin University, 71 Xinmin Street, Changchun, 130021 Jilin China

**Keywords:** Anti-gamma-aminobutyric acid-B receptor encephalitis, Limbic encephalitis, Autoantibody, Convulsive status epilepticus, Prognosis

## Abstract

**Objective:**

To investigate the clinical characteristics and prognosis of anti-gamma-aminobutyric acid-B (GABA-B) receptor encephalitis.

**Methods:**

This retrospective study enrolled nineteen patients with anti-GABA-B receptor encephalitis. Clinical manifestations, radiological and electroencephalogram features, treatment and outcomes were collected and analyzed. The neurological function was evaluated according to the modified Rankin Scale (mRS).

**Results:**

There were eleven patients in the favorable-prognosis group (mRS ≤ 2) and eight patients in the poor-prognosis group (mRS > 2). In the favorable-prognosis group, clinical symptoms included memory deterioration (*n* = 10; 90.9%), epileptic seizures (*n* = 9; 81.8%), psychiatric disorders (*n* = 9; 81.8%), and conscious disturbance (*n* = 5; 45.5%); magnetic resonance imaging (MRI) indicated an involvement of the limbic system in three (27.3%) cases in this group. Lung cancer was detected in one patient (9.1%). After an average follow-up period of 11.7 months, four (36.4%) patients were cured, and seven (63.6%) patients showed significant improvements. In the poor-prognosis group, all patients presented with memory deterioration, epileptic seizures, psychiatric disorders, and conscious disturbance; five (62.5%) patients had convulsive status epilepticus, and five (62.5%) patients developed respiratory failure; MRI indicated an involvement of the limbic system in seven (87.5%) cases. Malignant tumors were detected in five (62.5%) patients. After an average follow-up period of 14.8 months, seven (87.5%) patients died and one (12.5%) patient remained dependent in daily life.

**Conclusions:**

The clinical manifestations of anti-GABA-B receptor encephalitis include epileptic seizures, cognitive impairment and psychiatric disorders. Patients with convulsive status epilepticus or respiratory failure have poor outcomes. In anti-GABA-B receptor encephalitis, limbic system involvement is associated with a poor prognosis in and radiological examinations can reflect disease progression. Early diagnosis and appropriate treatment should be highlighted.

## Introduction

Limbic encephalitis (LE) refers to an acute or subacute inflammatory disorder of the central nervous system that predominantly affects the structures of the limbic system, such as the hippocampus, amygdaloid nucleus, insula and cingulate gyrus [1]. Clinically, LE is characterized by the impairment of recent memory, mental and behavioral disorders and epileptic seizures. Previously, LE was considered to be a paraneoplastic disease related to lung cancer, testicular cancer and some other tumors; thus, it was also known as paraneoplastic limbic encephalitis (PLE) [2, 3]. With the discovery of antibodies to onconeural intracellular antigens, such as Hu, PNMA2 (Ma2/Ta) and CV2/CRMP5, LE has been recognized as an autoimmune disease. Subsequently, antibodies against neuronal surface antigens, such as the gamma-aminobutyric acid-B (GABA-B) and N-methyl-D-aspartate (NMDA) receptors have been discovered and the cases of LE that are associated with these antibodies have been classified as a new-type limbic encephalitis or autoimmune synaptic encephalitis [4, 5].

Anti-GABA-B receptor encephalitis is an autoimmune encephalitis caused by antibodies to GABA-B receptor in the limbic system. This form of the disease accounts for approximately 5% of all cases of LE [4]. Recently, anti-GABA-B receptor encephalitis has gained great attention and an increasing number of cases have been reported in western countries. However, anti-GABA-B receptor encephalitis has rarely been studied in the Chinese population, and the relevant clinical and prognostic features in this region remain poorly understood. This study aimed to investigate the clinical characteristics and prognosis of anti-GABA-B receptor encephalitis in Northeast China.

## Materials and methods

### Patients

This retrospective study enrolled nineteen patients that had been diagnosed with anti-GABA-B receptor encephalitis at the Department of Neurology of the First Hospital of Jilin University between September 2014 and August 2017. The inclusion criteria were as follows: 1) acute or subacute onset (clinical course < 3 months) with progressive symptoms; 2) clinical symptoms consistent with the diagnosis of LE; 3) cerebrospinal fluid (CSF) examination results of slightly increased lymphocyte count or normal leucocyte count; 4) brain magnetic resonance imaging (MRI) revealed normal results or demonstrated abnormal signals in unilateral or bilateral limbic system; 5) anti-GABA-B receptor antibodies were positive in the serum and/or CSF; and 6) there were no infectious, traumatic, toxic, metabolic, intracranial neoplastic or demyelinating diseases.

This study was approved by the local Ethics Committee. Written informed consent was obtained from each participant.

### Data collection

The data, including clinical manifestations, radiological and electroencephalogram (EEG) features, laboratory examination results, treatment and outcomes, were collected and analyzed. The neurological function was evaluated according to modified Rankin Scale (mRS), and the patients were divided into two groups: a favorable-prognosis group (mRS ≤ 2) and a poor-prognosis group (mRS > 2) [6]. All of the patients underwent laboratory examinations for anti-autoimmune encephalitis antibodies, routine CSF testing, biochemical CSF testing, and EEG. Antibodies to the GABAB receptor, NMDA receptor, AMPA receptor, LGI1 and CASPR2 and classic paraneoplastic autoantibodies (anti-Hu, −Yo, −Ri, −CV2/CRMP5, −amphiphysin and - Ma2/Ta) in serum and/or CSF samples were measured using indirect immunofluorescence assay (Euroimmun AG, Lübeck, Germany). The titers of antibodies were categorized as slightly positive (+; < 1:10), positive (++; < 1:100), and strongly positive (+++; ≥1:100). Eighteen patients underwent brain MRI and pulmonary computed tomography (CT). Two patients underwent positron emission tomography-computed tomography (PET-CT), and two patients underwent pathological biopsy of the pulmonary lesion. Fifteen patients received immunomodulating therapy after the diagnosis of anti-GABA-B receptor encephalitis.

### Statistical analysis

SPSS 24.0 software (IBM Corp., Armonk, NY, USA) was used for statistical analyses. Continuous variables were expressed as the mean ± standard deviation (SD) and compared using Student’s t test. Categorical values were expressed as frequencies (percentage, %) and compared using Fisher’s exact test. *P* values of less than 0.05 were considered statistically significant and *P* values of less than 0.01 were considered highly significant.

## Results

### Clinical characteristics

Ten males and nine females, with an average age of 58.63 ± 11.43 years (range 23–79 years), were included in this study. Eighteen patients were Han Chinese and one patient was Chinese Korean. The symptom duration ranged from 14 h to 3 months. There were eleven patients in the favorable-prognosis group (mRS ≤ 2) and eight patients in the poor-prognosis group (mRS > 2). There was no significant difference in patients' age between the two groups (*P* = 0.305).

In the favorable-prognosis group, clinical symptoms included memory deterioration (*n* = 10; 90.9%), epileptic seizures (*n* = 9; 81.8%), psychiatric disorders (*n* = 9; 81.8%), and conscious disturbance (*n* = 5; 45.5%); In the poor-prognosis group, all of the patients presented with memory deterioration, epileptic seizures, psychiatric disorders, and conscious disturbance; five (62.5%) patients had convulsive status epilepticus, and five (62.5%) patients developed respiratory failure. Statistical analyses show a significant difference in the occurrence of convulsive status epilepticus and respiratory failure (*P* = 0.005). The detailed clinical characteristics of the nineteen patients with anti-GABA-B receptor encephalitis are summarized in Table 1.

**Table 1:** Clinical characteristics of nineteen patients with anti-GABA-B receptor encephalitis

Characteristics	Favorable-prognosis group (*n* = 11)	Poor-prognosis group (*n* = 8)
Gender (male/female)	5/6	5/3
Age (years)	56.27 ± 13.76	61.87 ± 6.66
Limbic system symptoms	10 (90.9%)	8 (100%)
Epilepsy/Convulsive status epilepticus	9 (81.8%)/0 (0%)	8 (100%)/5 (62.5%)
Psychiatric disorder	9 (81.8%)	8 (100%)
Conscious disturbance	5 (45.5%)	8 (100%)
Respiratory failure	0 (0%)	5 (62.5%)

### Radiological examinations

In the favorable-prognosis group, MRI revealed an involvement of the limbic system in three (27.3%) cases; the lesions were small and the distribution was restricted to the temporal lobe and hippocampus. In the poor-prognosis group, MRI revealed involvement of the limbic system in seven (87.5%) case. In these cases, the lesions were extensively distributed in the temporal lobe, insula and hippocampus. The lesions appeared hypointense on T1-weighted imaging and hyperintense on T2-weighted, fluid attenuated inversion recovery (FLAIR), and diffusion-weighted imaging. Statistical analysis shows that an involvement of the limbic system was more prevalent in the poor-prognosis group (*P* = 0.025). The detailed results are presented in Table 2 and Table 3.

**Table 2:** Radiological features of eighteen patients with anti-GABA-B receptor encephalitis

Group	Favorable-prognosis group (*n* = 10)	Poor-prognosis group (*n* = 8)	Total (*n* = 18)
Involvement of limbic system on MRI	3 (30.0%)	7 (87.5%)	10 (55.6%)
No involvement of limbic system on MRI	7 (70.0%)	1 (12.5%)	8 (44.4%)
Total	10 (100%)	8 (100%)	18 (100%)

*P* = 0.025 in Fisher’s exact test

**Table 3:** Initial symptoms, magnetic resonance imaging and electroencephalogram of nineteen patients with anti-GABA-B receptor encephalitis

Case No.	Initial symptoms	Magnetic resonance imaging	Electroencephalogram
Favorable-prognosis group			
1	Seizures	Abnormal signals in the right temporal lobe	Focal seizures followed by non-convulsive status epilepticus
2	Seizures, psychiatric abnormalities	Abnormal signals in the bilateral hippocampus and frontal lobes	Focal seizures originating from the temporal area
3	Seizures, psychiatric abnormalities	Normal	Non-convulsive status epilepticus manifesting as periodic lateralized epileptiform discharges
4	Seizures, psychiatric abnormalities	Not available	Normal
5	Seizures, psychiatric abnormalities	Leukoaraiosis	Focal seizures originating from the left temporal area
6	Seizures, cognitive impairment	Normal	Normal
7	Seizures	Normal	Sharp and sharp-slow waves originating from the right frontal-temporal area
8	psychiatric abnormalities, cognitive impairment	Abnormal signals in the left temporal lobe	Irregular slow waves originating from the frontal-temporal area
9	Seizures	Normal	Non-convulsive status epilepticus originating from the frontal midline area
10	Memory impairment	Leukoaraiosis and multiple lacunar infarctions	Scattered slow waves
11	Seizures	Normal	Focal seizures originating from the right frontal area
Poor-prognosis group			
12	Seizures	Abnormal signals in the right temporal lobe	Focal seizures originating from the left temporal-occipital area
13 (primary)	Seizures	Abnormal signals in the left thalamus, insula, temporal lobe, hippocampus, and bilateral frontal-parietal lobes	Focal status epilepticus originating from the left temporal area
13 (recurrence)	Seizures	Abnormal signals in the right hippocampus and temporal lobe	Focal status epilepticus originating from the right temporal area
14	Diarrhea, seizures	White matter demyelination and multiple lacunar infarctions	Slow waves originating from the frontal midline area
15	Seizures	Abnormal signals in the left temporal lobe	Focal seizures originating from the frontal area
16	Seizures	Abnormal signals in the right hippocampus	Focal status epilepticus originating from the right temporal area
17	Seizures	Abnormal signals in the bilateral hippocampus	Focal seizures originating from the left temporal area
18	Seizures	Abnormal signals in the bilateral hippocampus and insula	Non-convulsive status epilepticus originating from the frontal area
19	Seizures, confusion	Abnormal signals in the left hippocampus	Focal status epilepticus originating from the left temporal area

PET-CT revealed a peripheral lung cancer with lymphatic metastasis in one patient in the favorable-prognosis group. In the poor-prognosis group, lung cancer was observed in four patients and a malignant mediastinal tumor was discovered in one patient. There was a significant difference in the occurrence of malignant tumors between the two groups (*P* = 0.041). The detailed results are summarized in Table 4.

**Table 4:** Concurrence of malignant tumors and anti-GABA-B receptor encephalitis

Group	Favorable-prognosis group (*n* = 11)	Poor-prognosis group (*n* = 8)	Total (*n* = 19)
Accompanied malignant tumor	1 (9.1%)	5 (62.5%)	6 (31.6%)
No malignant tumor	10 (90.9%)	3 (37.5%)	13 (68.4%)
Total	11 (100%%)	8 (100%)	19 (100%)

*P* = 0.041 in Fisher’s exact test

**Table 5:** Laboratory tests, treatment and prognosis of nineteen patients with anti-GABA-B receptor encephalitis

Case No.	Anti-GABA-B receptor antibodies		Other antibodies		CSF routine		Treatment				Follow-up period (months)	Outcomes	Initial mRS on admission	Follow-up mRS
	Serum	CSF	Serum	CSF	WBC (× 10^6^/L)	Protein (g/L)	ICU	Latency to immuno-therapy (days)	Immunotherapy	Antiepileptic therapy				
Favorable-prognosis group														
1	+	+	–	–	8	0.36	+	70	MP	LEV	18	Cured	5	0
2 (primary)	++	++	–	–	18	0.57	+	30	IVIG	LEV				
2 (recurrence)	+	+	–	–	18	0.74	–	7	IVIG+MP	–	17	Improved	4	1
3	+	+	–	–	5	0.43	–	30	IVIG+MP	–	2	Improved	5	1
4	+++	+++	–	–	103	0.53	–	16	MP	–	27	Improved	2	1
5	++	++	–	–	42	0.39	+	18	IVIG	OXC	20	Cured	5	0
6	+	+	–	–	4	0.44	–	15	IVIG+MP	VPA + LEV	8	Improved	2	1
7	+	+	–	–	19	0.34	–	22	IVIG	–	5	Cured	2	0
8	+	+	–	NMDAR+	7	0.39	–	–	–	–	3	Improved	4	1
9	+	+	–	–	8	0.33	–	35	MP	CBZ	3	Improved	5	1
10	+	+	–	–	34	1.09	–	17	IVIG	–	2	Improved	2	1
11 (primary)	++	+	–	–	20	0	+	29	MP	OXC				
11 (recurrence)	+	+	–	–	6	0.24	–	–		LEV	27	Cured	3	0
Poor-prognosis group														
12	+	+	Hu+	–	43	0.39	+	15	IVIG	LEV	9	Death	4	6
13 (primary)	+~++	+~++	CV2/CRMP5++	CV2/CRMP5+++	9	0.63	+	15	IVIG+MP	LEV				
13 (recurrence)	+	+	CV2/CRMP5+	CV2/CRMP5+	9	0.63	+	8	IVIG+MP	–	13	Death	5	6
14	+++	+++	Hu+	Hu+	96	0.37	+	–	–	–	27	Death	5	6
15	+++	++	–	–	7	0.37	+	12	IVIG+MP	OXC	25	Death	5	6
16	+	+	–	–	13	0.39	+	–	–	–	12	Death	5	6
17	++	++	–	–	21	0.49	+	13	IVIG+MP	LEV	20	Death	5	6
18	+	+	–	–	13	0.29	+	–	–	LEV	3	Death	5	6
19	+	+	–	–	31	0	+	5	IVIG+MP	OXC	9	Unchanged	5	5

*CSF,* cerebrospinal fluid; *WBC,* white blood cell; *mRS,* modified Rankin scale; *NMDAR,* anti-N-methyl-D-aspartate receptor antibody; *Hu,* anti-Hu antibody; *CV2/CRMP5,* anti-CV2/CRMP5 antibody; *MP,* methylprednisolone; *IVIG,* intravenous immunogloblin; *LEV,* levetiracetam; *OXC,* oxcarbazepine; *VPA,* valproic acid; *CBZ,* carbamazepine

### EEG examinations

In the favorable-prognosis group, epileptic abnormal discharges were noted in seven cases, including focal seizures in five cases and non-convulsive status epilepticus in three cases; nonspecific irregular slow waves were observed in two cases, and the EEG results were normal in two cases. In the poor-prognosis group, epileptic abnormal discharges were observed in seven cases, including focal seizures or focal status epilepticus in six cases and non-convulsive status epilepticus in one case; nonspecific irregular slow waves were observed in one case. The detailed EEG results are presented in Table 3.

### Laboratory tests

All patients were positive for serum anti-GABA-B receptor antibodies. The titers of anti-GABA-B receptor antibodies in the CSF ranged from slight positivity to strong positivity. There was no significant difference in the CSF antibody titer between the two groups (*P* = 0.442). In the favorable-prognosis group, one case showed positivity for the anti-NMDAR antibody. In the poor-prognosis group, anti-Hu antibody positivity in the serum and CSF was observed in two patients, and anti-CV2/CRMP5 antibody positivity was observed in the serum and CSF in one patient. There was no significant difference in the observation of anti-neuron antibody positivity between the two groups (*P* = 0.262).

The CSF appearance was colorless and clear in all patients. The pressure ranged from 70 mmH_2_O to 300 mmH_2_O. In the favorable-prognosis group, an elevated leukocyte count was found in six cases and an elevated protein level was noted in three cases; in the poor-prognosis group, an elevated leukocyte count was noted in seven cases and an elevated protein level was noted in two cases. There was no significant difference in the leukocyte count (*P* = 0.177) or the protein level (*P* > 0.05) between the two groups. The detailed results are presented in Table 5.

### Treatment and prognosis

In the favorable-prognosis group, ten patients underwent immunomodulating therapy, four patients were treated with methylprednisolone (120~1000 mg), four patients were treated with intravenous immunogloblin (IVIG; 0.4 g/kg/day), two patients received combined methylprednisolone and IVIG treatment, and no specific treatment was performed in one patient. After an average follow-up period of 11.7 months (range, 2~27 months), four (36.4%) patients were cured, and seven (63.6%) patients showed significant improvement. After treatment with antiepileptic drugs, the epileptic symptoms were well controlled in all patients.

In the poor-prognosis group, one patients were treated with methylprednisolone and succumbed to respiratory failure; no specific treatment was performed in three patients, all of whom succumbed to respiratory failure; four patients received combined methylprednisolone and IVIG treatment, all of whom had concomitant lung cancer, and three of these patients succumbed to lung cancer progression. Overall, after an average follow-up period of 14.8 months, seven (87.5%) patients had died and one (12.5%) patient remained dependent on care in daily life. In five patients, the epilepsy responded poorly to antiepileptic drugs. The detailed clinical profiles are summarized in Table 5.

## Discussion

Anti-GABA-B receptor encephalitis is a rare autoimmune LE [7]. As previously reported, the average onset age of anti-GABA-B receptor encephalitis is 60~70 years, with no obvious gender preference. The clinical symptoms of this disease include epileptic seizures, memory impairment, anxiety, conscious disturbance, and disorientation [7]. In some cases, anti-GABA-B receptor encephalitis can also manifest as oblique clonus, myoclonus, Stiffman syndrome, and cerebellar ataxia. Notably, most patients with anti-GABA-B receptor encephalitis have concomitant small-cell lung cancer [7–11]. Until now, anti-GABA-B receptor encephalitis has rarely been reported in the Chinese population. In the current study, we retrospectively analyzed the clinical characteristics and prognosis of nineteen patients with anti-GABA-B receptor encephalitis in Northeast China. In the literature, elderly age is associated with poor prognosis in this disease, which may be related to the high incidence of malignancy in the elderly [7–9, 12, 13]. In our cohort, the average age of onset was 58.63 ± 11.43 years, which is consistent with the onset ages that have been reported previously; nevertheless, we did not find any correlation between patient age and prognosis. Additionally, in this study, 89% patients presented with epilepsy as an onset symptom, 94% patients presented with limbic system symptoms, such as memory deterioration and disorientation, and 89% patients presented with psychobehavioral disorders. These findings were consistent with those of previous reports, in which the incidence of epilepsy in anti-GABA-B receptor encephalitis was 80%~ 100% and epilepsy could rapidly develop into status epilepticus [7–9, 12, 13].

Since the discovery of GABA-B in 1950, it has been considered to be the major inhibitory neurotransmitter in the brain, playing an important role in the maintenance of the balance between neuronal excitation and inhibition [14]. GABA-B receptor activation can limit the duration of hyperactivity of a neuronal network and prevent excessive synchronization of neuronal activities [15]. It has been speculated that epileptic seizures are caused by an imbalance between neuron excitation and inhibition, due to the interruption of neurosynaptic function secondary to the existence of anti-GABA-B receptor antibodies. In the current study, seventeen (89%) of the included patients had epilepsy, including generalized tonic-clonic seizure, focal secondary generalized seizure, and focal seizures with or without unconsciousness. In the literature, the incidence of status epilepticus following anti-GABA-B receptor encephalitis was approximately 10%~ 25% [8]. Consistently, five (26%) of the patients included in this study had convulsive status epilepticus, all of whom had poor outcomes. Notably, in the poor-prognosis group, five patients developed respiratory failure, which needed assistant treatment with mechanical ventilation. These findings indicate that convulsive status epilepticus and respiratory failure may be predictors for poor prognosis in anti-GABA-B receptor encephalitis. Additionally, one patient presented with diarrhea as the onset symptom. As previously reported, LE can lead to autonomic dysfunction via the connection between the medial temporal lobe and insular cortex [16, 17]. Loftspring et al.*,* reported a case of anti-GABA-B receptor encephalitis with cardiac autonomic dysfunction [18]. However, gastrointestinal autonomic dysfunction has not yet been reported in patients with anti-GABA-B receptor encephalitis. Thus, the current study has added to the known symptom spectrum of this disease.

Although epileptic seizures are the major clinical manifestation of anti-GABA-B receptor encephalitis, the EEG features are usually non-specific. In the favorable-prognosis group of this study, epileptic abnormal discharges were noted in seven cases. Such abnormalities include ictal or interictal sharp-slow waves and spike-slow waves, more than half of which showed focal seizures that originated in frontal and temporal regions. In the poor-prognosis group, seven patients had epileptic abnormal discharges, and six of these manifested as focal seizures or focal status epilepticus originating in frontal and temporal regions. Notably, four patients had non-convulsive status epilepticus. Non-convulsive status epilepticus refers to an extreme cerebral dysfunction characterized by continuous or repeated epileptic waves over 30 min. Previous studies have found that elderly patients with non-convulsive status epilepticus had a poor prognosis, which may be related to the more serious clinical severity and nosocomial infection [19]. Recently, the relationship between anti-GABA-B receptor encephalitis and non-convulsive status epilepticus has gained more attention, while the correlation between non-convulsive status epilepticus and clinical prognosis remains unclear. In our study, there was no significant difference in the occurrence of non-convulsive status epilepticus between the two groups, indicating that non-convulsive status epilepticus is not correlated with the prognosis of anti-GABA-B receptor encephalitis.

Since the discovery of the NMDAR antibody in 2007, there have been several reports of novel anti-neuronal antibodies related to autoimmune encephalitis. These antibodies can be divided into two categories: 1) antibodies against neuronal surface receptors, including anti-NMDAR antibodies, anti-GABA-B receptor antibodies, anti-LGI-1 antibodies, anti-α-amino-3-hydroxy-5-methyl-4-isoxazolepropionic acid receptor (AMPAR) antibodies, and anti-voltage-gated potassium channels (VGKC) antibodies; and 2) antibodies against intracellular antigens, such as anti-Hu antibodies and anti-Ma2 antibodies. Generally, autoantibodies have strong specificity and diagnostic values. Co-expression of multiple antineuronal autoantibodies is rare in autoimmune encephalitis, but is relatively more common in anti-GABA-B receptor encephalitis with an incidence of 7%~ 40% [7, 8, 11, 12]. In a previous study involving 20 patients with anti-GABA-B receptor encephalitis, positivity for multiple antibodies was observed in seven (35%) cases; five patients with concomitant small-cell lung cancer were found to be positive for the paraneoplastic antibodies SOX1 and Ri/ANNA2, and in two patients that did not have small-cell lung cancer the GAD65 and anti-NMDAR antibodies were detected. In our study, although positivity for the anti-NMDAR antibody, anti-CV2/CRMP5 antibody, and anti-Hu antibody were detected in a few cases, there was no significant difference in the autoantibodies that were detected between the two groups. Further research is needed to understand the clinical significance of these paraneoplastic antibodies in anti-GABA-B receptor encephalitis [8].

**Fig. 1:**
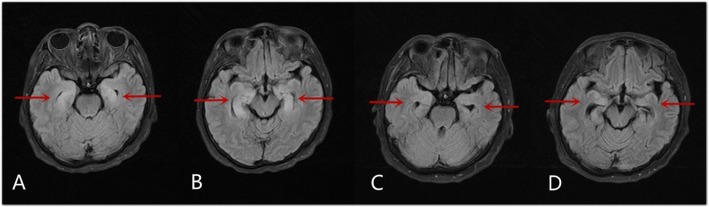
Magnetic resonance imaging of Case two. **a**-**b** Magnetic resonance imaging (fluid-attenuated inversion recovery sequence; FLAIR) shows abnormal signals in the bilateral hippocampus, as indicated by the red arrows. **c**-**d** Repeated magnetic resonance imaging 7 months later shows significantly improvement

**Fig. 2:**
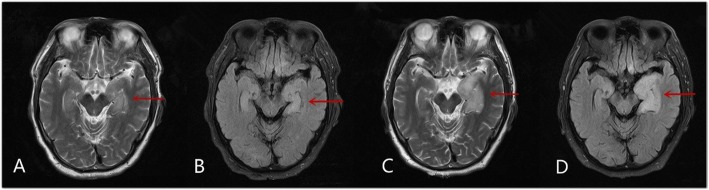
Magnetic resonance imaging of Case eight. **a**-**b** Magnetic resonance imaging (FLAIR) shows abnormal signals in the left hippocampus, as indicated by the red arrow. **c**-**d** One month later, the clinical symptoms of the patient were aggravated, and repeated magnetic resonance imaging shows an enlargement of the lesion

**Fig. 3:**
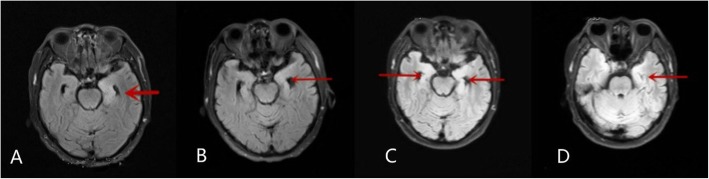
Magnetic resonance imaging of Case nineten. **a** On admission, magnetic resonance imaging (FLAIR) shows abnormal signals in the left hippocampus, as indicated by the red arrow. **b** The patient was treated with gamma globulin and methylprednisolone. Following treatment, repeated magnetic resonance imaging shows the signal abnormality was alleviated. **c**-**d** Two months later, the patient developed severe memory deterioration. Magnetic resonance diffusion-weighted imaging shows that an enlargement of the lesion to also effect the right hippocampus, as indicated by the additional red arrow

The radiological features of anti-GABA-B receptor encephalitis are non-specific and may be negative in some patients [7, 9, 12]. We found that involvement of the limbic system, as determined through MRI, was more common in the poor-prognosis group than in the favorable prognosis group. Thus, we speculate that MRI manifestations can reflect the disease progression in this disease, to a certain degree. For instance, the patient in Case two presented with a 22-day history of seizures and balderdash, in which the brain MRI results revealed hyperintensities in the bilateral hippocampus and right frontal lobe; the patient was clinically and radiologically improved after gamma globulin treatment (Fig. 1). The patient in Case eight presented with memory deterioration, and the brain MRI results revealed a hyperintensity in the left hippocampus; in the following 1 month after admission, the symptom was aggravated, and repeated MRI showed that the lesion had grown (Fig. 2). The initial MRI in Case nineteen showed abnormal signals in the left hippocampus. And, although, the patient was clinically and radiologically improved after treatment with gamma globulin and methylprednisolone, 3 months after discharge, the patient was readmitted due to memory deterioration, and repeated brain MRI showed an enlargement of the lesion (Fig. 3).

The optimal treatment for anti-GABA-B receptor encephalitis remains debated. Mainstream therapeutic strategies include immunomodulating therapy, immunosuppressive therapy, tumor resection, and chemotherapy. Current immunotherapies include IVIG, plasmapheresis, corticosteroids, and cyclophosphamide. Previous evidence showed early immunotherapy could significantly improve the clinical prognosis and lower the mortality [20]. However, in our study, there was no significant difference in the effects of immunotherapy between two groups. Further clinical trials are required to determine the definitive efficacy of immunotherapy. Additionally, epileptic seizures in patients with anti-GABA-B receptor encephalitis are usually refractory, and the treatment is challenging [21]. When conventional antiepileptic drugs provide no benefits, immunotherapy may be helpful in the treatment of the epileptic symptoms [22]. In our study, epilepsy was well controlled in the favorable-prognosis group as well as the poor-prognosis group following immunotherapy. Therefore, we recommend immunotherapy as the first-line treatment for anti-GABA-B receptor encephalitis. In recent years, the concept of autoimmune limbic encephalitis (ALE) has been gradually accepted. This term refers to new-onset seizures in the context of acute or subacute neurocognitive impairment. As mentioned above, although limbic encephalitis can present with a variety of neuropsychiatric symptoms, seizures remain the most common symptoms and often progress to refractory ALE. Beyond the use of antiepileptic drugs and treatment for potential tumors, there is no effective treatment for ALE [20]. Previous studies have shown that immunomodulatory therapy may be an effective treatment when epileptic seizures respond poorly to conventional antiepileptic drugs [21]. These findings further support the idea that immunomodulatory therapy should be the first-line treatment for anti-GABA-B receptor encephalitis. In this study, the patients were treated with antiepileptic drugs, according to individual seizure type and EEG results. In the favorable-prognosis group, the seizures were well controlled.

The prognosis of anti-GABA-B receptor encephalitis remains unclear. Generally, early diagnosis and timely intervention are associated with a better prognosis, although some patients may die due to the poor response to treatment [23]. In this study, we found that patients with status epilepticus, respiratory failure and/or an involvement of the limbic system had worse outcomes than patients without these features. We speculate that these factors may be predictors of poor prognosis.

## Conclusions

Anti-GABA-B receptor encephalitis clinically manifests as epileptic seizures, cognitive impairment and psychiatric disorders. Patients with convulsive status epilepticus or respiratory failure have poor outcomes, and the involvement of the limbic system is associated with a poor prognosis. Radiological examinations can reflect the disease progression and prompt diagnosis and appropriate treatment should be emphasized.

## Data Availability

All data used and/or analyzed during the study is available on request from the corresponding author.

## Abbreviations

ALE: autoimmune limbic encephalitis
AMPAR: α-amino-3-hydroxy-5-methyl-4-isoxazolepropionic acid receptor
CASPR2: contactin-associated protein-like 2
CBZ: carbamazepine
CSF: cerebrospinal fluid
CT: computed tomography
CRMP5: collapsin response mediator protein 5
EEG: electroencephalogram
FLAIR: fluid attenuated inversion recovery
GABA-B: gamma-aminobutyric acid-B
IVIG: intravenous immunoglobulin
LE: limbic encephalitis
LEV: levetiracetam
LGI1: leucine-rich glioma-inactivated 1
MP: methylprednisolone
MRI: magnetic resonance imaging
mRS: modified Rankin Scale
NMDA: N-methyl-D-aspartate
NMDAR: N-methyl-D-aspartate receptor
OXC: oxcarbazepine
PET-CT: positron emission tomography-computed tomography
PLE: paraneoplastic limbic encephalitis
SD: standard deviation
VGKC: voltage-gated potassium channels
VPA: valproic acid
WBC: white blood cell
